# Myopericarditis and exertional rhabdomyolysis following an influenza A (H3N2) infection

**DOI:** 10.1186/1471-2334-13-283

**Published:** 2013-06-21

**Authors:** Guo-Shiang Tseng, Chung-Yueh Hsieh, Ching-Tsai Hsu, Jung-Chung Lin, Jenq-Shyong Chan

**Affiliations:** 1 Department of Internal Medicine, Taoyuan Armed Forces General Hospital, Taoyuan County, Taiwan; 2 Division of Infectious Diseases and Tropical Medicine, Tri-Service General Hospital, National Defense Medical Center, Taipei, Taiwan; 3 Renal Division, Department of Internal Medicine, Taoyuan Armed Forces General Hospital, No.168, Jhongsing Road, Longtan Township, Taoyuan County, 32551, Taiwan

**Keywords:** Myopericarditis, Exertional rhabdomyolysis, Influenza A, Inflammation

## Abstract

**Background:**

Acute myopericarditis and exertional rhabdomyolysis, two uncommon but well-described diseases with potentially life-threatening effects, are generally considered as independent clinical entities. However, they may in fact be pathophysiologically related under certain circumstances. This is the first ever report of influenza myopericarditis provoked by exertional rhabdomyolysis to the best of our knowledge.

**Case presentation:**

A 25-year-old immunocompetent Chinese man presented with bilateral leg pain, dizziness, and shortness of breath on admission soon after completing vigorous training comprising running drills. Exertional rhabdomyolysis was diagnosed with 44 fold high serum creatine phosphokinase. Then he developed chest pain, pericardial effusion, changes of electrocardiography and positive troponin I suggestive of myopericarditis. Influenza A (H3N2) virus infection was confirmed by polymerase chain reaction analysis of nasopharyngeal wash samples. Other possible infective and autoimmune causes were excluded. Patient recovered completely with anti-inflammatory therapy and the supportive care.

**Conclusion:**

This case suggests that clinicians who treat patients with exertional rhabdomyolysis should be aware of the potential vulnerability to acute myopericarditis, especially in the presence of recent influenza A infection.

## Background

Acute myopericarditis and exertional rhabdomyolysis, two uncommon but well-described diseases with potentially life-threatening effects, are generally considered as independent clinical entities. However, they may in fact be pathophysiologically related under certain circumstances. Here, we report an unusual case of an immunocompetent man who presented to the emergency department after a resolving upper respiratory infection with an unusual presentation of exertional rhabdomyolysis accompanied by acute myopericarditis caused by influenza A. The relationship among these three pathologies and their clinical implications are discussed.

## Case presentation

A previously healthy 25-year-old male conscript presented to our emergency department. He complained of bilateral leg pain, dizziness, and shortness of breath soon after completing vigorous training comprising running drills. Ten days before admission, he attended his family clinic complaining of cough and a sore throat. The rapid test for influenza A was positive and he was treated with a 5-day course of oseltamivir. Five days after completing the course, his symptoms had resolved completely and he recommenced regular military training. There was no history of trauma or illicit drug use. He also denied having fatigue, weakness, or weight loss, and the rest of his verbal review of systems was unremarkable.

**Figure 1:**
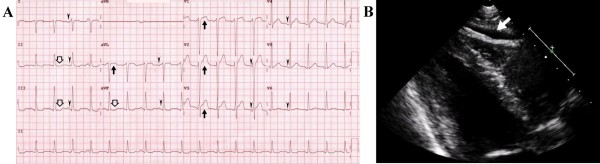
**Myopericarditis associated with influenza A infection and exertional rhabdomyolysis. A**: Electrocardiogram showing diffuse PR segment depression (arrowheads) and localized concave upward ST segment elevation in leads aVL and V1–V3 (black arrows) with reciprocal changes in the inferior leads (hollow arrows). **B**: Parasternal long-axis view on transthoracic echocardiography demonstrating a moderate-sized pericardial effusion (white arrow).

Physical examination revealed the following values: temperature, 38.2°C; heart rate, 154 beats/min; respiratory rate, 26 breaths/min; and blood pressure, 93/48 mmHg. The results of his physical examination were otherwise unremarkable except for tenderness on palpation of the muscle groups of the thighs. Of note, there were no neurological deficits, and heart sounds showed no definite gallop rhythm. Laboratory investigations revealed the following values: creatine phosphokinase (CPK), 5,555 U/L with an MB fraction of 78 ng/mL; hemoglobin, 17.2 g/dL; platelet count, 348,000 cells/mm^3^; and total white blood cell (WBC) count, 15,840 cells/mm^3^ with a differential of 32% lymphocytes, 4% monocytes, 58% neutrophils, and 3% bands. The cardiac troponin I (cTnI) level was 0.46 μg/L (reference < 0.1 μg/L) and the lactate dehydrogenase level was 836 U/L (reference 135–225 U/L). His serum creatinine level was 2.3 mg/dL and his serum electrolyte levels were normal. Liver function tests indicated an elevated aspartate aminotransferase concentration of 47.8 U/L and alanine aminotransferase concentration of 58 U/L; total bilirubin, alkaline phosphatase, total protein, and albumin levels were all within normal limits. The result of a urine toxicology screen was negative. His urine dipstick test was positive for blood with a pH of 6.5 but no red blood cells or WBCs were observed by microscopy. A test for urine myoglobin had a positive result. The results of a coagulation test, thyroid function test, complement test and antinuclear antibody test were normal. The C-reactive protein level and erythrocyte sedimentation rate were initially slightly elevated but had normalized 7 days later. Cultures of blood samples tested negative, and viral studies were negative apart from detecting influenza A (H3N2) virus by polymerase chain reaction analysis of nasopharyngeal wash specimens. Chest radiography did not show any abnormalities. Electrocardiography revealed a regular narrow QRS complex but diffuse PR segment depression and localized concave upward ST segment elevation in leads aVL and V1–V3 with reciprocal changes in the inferior leads (Figure 1A). Echocardiography disclosed normal systolic function without pericardial effusion or valve regurgitation.

In the 3 days after admission, the CPK peaked at 34,308, and the cTnI and creatinine peaked at 1.17 and 2.46, respectively. He also developed new-onset left-sided and central chest pain. The pain was pleuritic in nature, and a pericardial friction rub was noted on auscultation. A second echocardiography demonstrated moderate circumferential pericardial effusion without any other abnormalities (Figure 1B), consistent with myopericarditis with a predominant pericarditic component. Based on these findings, he was treated with aspirin for acute myopericarditis and aggressive hydration and urinary alkalinization as per the local rhabdomyolysis protocol. After a total of 20 days of hospitalization, the patient’s leg pain resolved, chest pain relieved, and CPK level declined to 656 U/L. His serum cTnI and creatinine levels normalized after anti-inflammatory therapy and supportive management. Preserved ejection fraction without deterioration of the pericardial effusion was noted in subsequent serial follow-up echocardiographic assessments. The patient was discharged with a normal electrocardiogram and no further sequelae.

## Discussion

Myopericarditis, or inflammation of the myocardium and pericardium, can present with an overlap of the symptoms of myocarditis (flu-like symptoms such as fever, fatigue and myalgias) and pericarditis (sharp or pleuritic chest pain that is relieved with sitting forward and worsened by laying back) [1]. In clinical practice both pericarditis and myocarditis coexist because they share familiar causative agents, mainly cardiotropic viruses (Table 1) [2-6]. Enteroviruses, especially group B coxsackieviruses, appear to be the major implicated agents. The term “myopericarditis” indicates a primarily “pericarditic syndrome” and it is responsible for the majority of cases. The inflammatory process is usually self-limited without overt sequelae and can occur as seasonal epidemics, especially coxsackieviruses and influenza. Nevertheless, preliminary studies have shown that strenuous exercise can markedly accelerate viral myopericarditis and enhance the inflammatory process [7,8].

**Table 1:** Etiologies of viral myopericarditis

**Common:**	**Uncommon:**
Coxsackievirus^*^	Parainfluenza
Adenovirus	Human immunodeficiency virus
Cytomegalovirus	Varicella
Echovirus	Mumps
Influenza^*^	Rubeola
Epstein–Barr virus	Rubella
Herpesvirus	Poliomyelitis
Hepatitis virus	Rhinovirus
Parvovirus	Vaccinia (smallpox vaccine)
	Variola
	Other

^*^ Viral myopericarditis is usually a self-limited disease and can occur as seasonal epidemics, especially coxsackievirus B and influenza.

Influenza is an acute febrile illness caused by influenza viruses. Most infections are uncomplicated and the illness is usually limited to symptoms of upper respiratory infection in combination with several constitutional symptoms, including headache, lethargy, and myalgia. However, some patients are at risk of severe illness and fatal complications affecting multiple organ systems [9]. Various complications of influenza A infection have been reported in the pulmonary, neurological, renal, cardiac and muscular systems [10-12]. Our patient presented with exertional rhabdomyolysis and concurrent myopericarditis, a rare combination. Reports in the literature have indicated that, in adults, exercise during or after a viral illness increases the risk of rhabdomyolysis [13]. As muscle damage occurs, release of constituents of necrotic muscle results in the accumulation of free radicals and tumor necrosis factor-α (TNF-α) in the serum, which are responsible for a systemic inflammatory reaction during rhabdomyolysis. Experimental and necropsy studies have shown convincingly that free radicals produced in any organ of the body can induce myocardial damage [14]. Proinflammatory cytokines including TNF-α are also considered important in the initiation and development of the pathophysiology of inflammatory cardiomyopathies [15]. Moreover, human studies have shown that infection of the myocardium by influenza A is associated with increased expression of TNF-α and its receptors in the myocardium [16]. These findings suggest that exertional rhabdomyolysis may be an important underlying condition for the development of cardiac involvement in influenza A infection.

**Figure 2:**
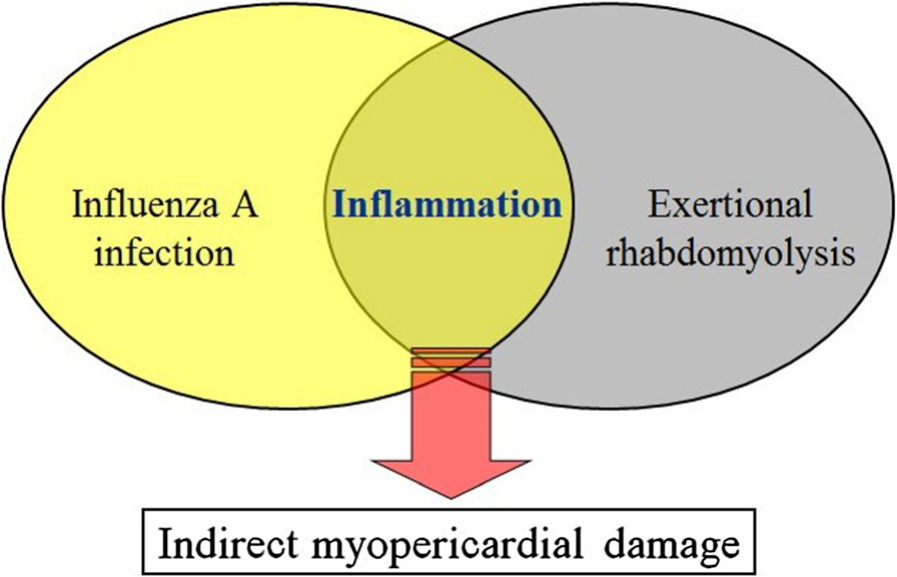
Rhabdomyolysis and influenza A infection may provoke an indirect inflammatory mechanism responsible for myopericardial damage.

Cardiac involvement in influenza occurs usually during the first week after the onset of influenza symptoms [17]. This may result from direct viral invasion or from the host immune-mediated inflammatory pathology; the immunological dissonance usually plays a major role in the later phases of infection, even in the absence of viral genome [1]. In our case, myopericardial damage occurred later in the course of infection, on the tenth day after the onset of influenza symptoms, and was associated with exertional rhabdomyolysis. More intriguingly, the patient’s clinical manifestations and echocardiographic images also progressed in parallel with the increase in CPK concentration. We consequently assume that this cardiac disorder was provoked by exertional rhabdomyolysis, which may be due to the profound injury caused by inflammatory mediators to the heart, predisposing it to develop influenza myopericarditis (Figure 2). Alternatively, there may be coincidence of both diseases with a time lag. Further studies are needed to elucidate the molecular mechanisms associated with these clinical phenomena.

## Conclusion

Exertional rhabdomyolysis with acute kidney injury and acute myopericarditis are uncommon yet potentially life-threatening complications of influenza A infection. To the best of our knowledge, this is the first case of influenza myopericarditis spurred by exertional rhabdomyolysis. It suggests that clinicians who treat patients with exertional rhabdomyolysis should be aware of the potential vulnerability to acute myopericarditis, especially in the presence of recent influenza A infection. Early diagnosis and the intensive care with supportive treatment of such cases can reduce the risk of further cardiac events. As influenza A affects millions of people worldwide, clinicians should also advise patients to avoid strenuous physical activities during and after the infection.

## Consent

Written informed consent was obtained from the patient for the publication of this case report. A copy of the written consent is available for review by the Editor-in-Chief of this journal.

## Abbreviations

CPK: Creatine phosphokinase; WBC: White blood cell; cTnI: Cardiac troponin I; TNF-α: Tumor necrosis factor-α.

## Competing interests

The authors declare that they have no competing interests.

## Authors’ contribution

All authors were involved in the management of the patient. GST and JSC researched the background literature on the case and wrote the first draft. CYH and JCL contributed towards the discussions and analysis of the case. CTH contributed to the clinical management of the patient. All authors read and approved the final manuscript.

## Pre-publication history

The pre-publication history for this paper can be accessed here:

http://www.biomedcentral.com/1471-2334/13/283/prepub
